# Hepatocellular carcinoma with hilar bile duct tumor thrombus versus hilar Cholangiocarcinoma on enhanced computed tomography: a diagnostic challenge

**DOI:** 10.1186/s12885-020-6539-7

**Published:** 2020-01-22

**Authors:** Xiaoqi Zhou, Jifei Wang, Mimi Tang, Mengqi Huang, Ling Xu, Zhenpeng Peng, Zi-Ping Li, Shi-Ting Feng

**Affiliations:** 1 Department of Radiology, The First Affiliated Hospital, Sun Yat-Sen University, 58 Zhongshan 2nd Road, Guangzhou, 510080 Guangdong China; 2 Faculty of Medicine and Dentistry, University of Western Australia, Perth, Australia

**Keywords:** Hepatocellular carcinoma, Hilar bile duct tumor thrombus, Hilar cholangiocarcinoma, Computed tomography

## Abstract

**Background:**

Hepatocellular carcinoma (HCC) with hilar bile duct tumor thrombus (HBDTT) often mimic hilar cholangiocarcinoma (hilar CC). The purpose of this study is to analyze the Computed Tomography (CT) characteristics of HCC with HBDTT and to identify imaging features to aid its differentiation from hilar CC on enhanced CT.

**Methods:**

We retrospectively identified 58 cases with pathologically proved HCC with HBDTT between 2011 and 2018. Seventy-seven cases of pathologically proven hilar CCs were selected during the same period. The clinical features and CT findings of the two groups were reviewed and compared.

**Results:**

HCC with HBDTTs are more commonly found in men (87.9% vs 63.6%, *p* = 0.001) with lower age of onset (49.84 vs 58.61 years; *p* < 0.001) in comparison to hilar CCs. Positive correlation were identified between HCC with HBDTTs and chronic HBV infection (72.4% vs 11.7%; *p* <  0.001), increased serum AFP (67.2% vs 1.3%; *p* <  0.001), CA19–9 level (58.6% vs 85.7%; *p* <  0.001) and CEA level (3.4% vs 29.9%; *p* = 0.001), parenchymal lesion with intraductal lesion (100% vs 18.2%; *p* <  0.001), washout during the portal venous phase (84.5% vs 6.5%; *p* <  0.001), thickened bile duct wall (8.6% vs 93.5%; *p* <  0.001), intrahepatic vascular embolus (44.8% vs 7.8%; *p* <  0.001), splenomegaly (34.5% vs 2.6%, *p* <  0.001). A scoring system consisting of the five parameters obtained from characteristics mentioned above was trialed. The sensitivity and specificity for diagnosing HCC with HBDTT were 96.39, 100 and 92.5% respectively when the total score was 2 or more.

**Conclusions:**

HCC with HBDTTs are often distinguishable from hilar CCs based on washout during portal venous phase without thickened bile duct wall. HBV infection and serum AFP level facilitate the differentiation.

## Background

Liver cancer is the sixth most common malignancy and the second leading cause of cancer death for males worldwide [1]. Hepatocellular carcinoma (HCC) is the most common type of primary liver cancer, comprising 75 to 85% of cases [1], but HCC with bile duct tumor thrombus (BDTT) is uncommon with incidence between 0.53 to 12.9% [2–6]. Previous studies have attempted to explore the clinical, pathological, imaging features as well as treatment and prognosis of HCC with BDTT [7–13]. Both CT and MRI have diagnostic value for HCC with BDTT and can evaluate the extension of tumor thrombus. It was speculated that HCC with BDTT are more invasive than HCC without BDTT, which might indicate a poor prognosis. However, although HCC can be treated in a variety of ways, long-term survival for HCC with BDTT are best achieved by surgical resection. Therefore, early diagnosis and surgical treatment are important to improve survival.

HCC with hilar bile duct tumor thrombus (HBDTT) is a common subtype of HCC with BDTT which can involve the left and right hepatic duct, common hepatic duct and common bile duct. Most of the HBDTT might mimic hilar cholangiocarcinoma (hilar CC) in clinical presentation because they share the same symptoms such as obstructive jaundice and upper abdomen pain. On the other hand, both HCC with BDTT and hilar CC have similar image features like hilar neoplasm, obstructed hilar bile duct and upstream bile duct dilatation [3, 9, 11, 14, 15]. However, it is important to distinguish HCC with HBDTTs from hilar CCs preoperatively as different surgical procedures are required. Partial hepatectomy is the main surgical option for HCC with HBDTT [11] while lobar hepatectomy, bile duct resection and Roux-en-Y hepaticojejunostomy are often necessary in hilar CC [16].

To our knowledge, no previous study has illustrated the radiological features to distinguish HCC with HBDTTs from hilar CCs. Thus, the purpose of our study is to describe the imaging characteristics of HCC with HBDTT on dynamic enhanced CT imaging to identify the helpful imaging features for differentiating it from hilar CC.

## Methods

### Patient population

This study was approved by the institutional review board of our institution, and patient informed consent was waived due to the retrospective nature of this study.

1827 patients underwent dynamic enhanced CT imaging with pathologically proved HCC post operatively were identified at our institute between January 2011 to August 2018. Further selection with key words “hepatocellular carcinoma” and “bile duct thrombus” identified 94 cases with pathologically confirmed HCC with BDTT. Out of these, 36 cases involved peripheral bile duct tumor thrombus and 58 cases of HCC with HBDTT. These 58 HCC with HBDTTs were subsequently enrolled in the study.

**Fig. 1:**
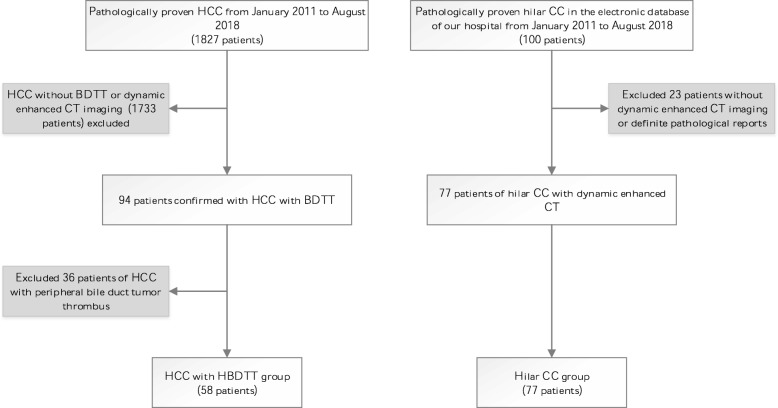
Flow diagram of study population

To establish a comparison group, a search using keywords “perihilar cholangiocarcinoma” or “hilar cholangiocarcinoma” in the electronic database of our hospital during the same study period revealed 100 patients. Twelve patients did not undergo preoperative CT examination, 1 patient with intrahepatic cholangiocarcinoma, 1 patient with high-grade intraepithelial neoplasia (CIN III) and 9 patients without pathological reports were excluded from the study. A total of 77 patients with pre-operative CT examination and post-operative pathological diagnosis were collected as the comparison group. A flow diagram for the study population is presented in Fig. 1.

### Clinical information

Electronic medical records of the 135 patients (58 HCC with HBDTTs patients and 77 hilar CC patients) were reviewed. Patient demographics, initial symptoms, hepatitis history, relevant serum tumor markers and preoperative diagnosis provided in the CT reports were compared and analysed. The levels of four serum tumor markers were recorded with a normal reference level of less than 20 μg/L for AFP, less than 35 U/mL for CA19–9, less than 35 U/mL for CA-125, and less than 5 μg/L for CEA.

### Image acquisition

135 patients from both two groups underwent biphasic CT including unenhanced phase, arterial phase, and portal venous phase. 64 Slice MDCT scanner (Toshiba, Aquilion, Japan) were used. The scanning was obtained along the craniocaudal plane, with the slice thickness: 0.5 mm, tube voltage: 120 kV, tube current 250 mA.

Nonionic contrast material (iopromide, Ultravist, Bayer Schering Pharma, Germany) was injected into an antecubital vein at a rate of 3.5–4.0 mL/s with power injector 1.5 mL/kg. Scan delay for the arterial phase and portal phase was 34–37 s, 60–70s.

### Image analysis

Two experienced abdominal radiologists (with 18 years and 15 years of experience respectively) reviewed the CT images in consensus. The readers were blinded to the number of patients in each patient category, to the clinical information, and to the histopathologic diagnoses, although they were aware that the study population consisted of patients with either HCC with HBDTT or hilar CC. All of the images were shuffled and randomly reviewed.

We evaluated the following findings: tumor characteristics including location, size, precontrast density and pattern of contrast enhancement; the presence of thickened bile duct walls; the presence of enlarged perihilar and retroperitoneum lymph nodes; the presence of vascular tumor thrombus; the presence of intrahepatic bile duct calculus; and the presence of extrahepatic manifestations of cirrhosis including splenomegaly, ascites and esophagogastric varices. Tumor location was divided as involving both liver parenchyma and bile duct or not. Tumor size of HCC with HBDTT was defined as the long diameter of the parenchyma lesion, and that of hilar CC was the axial diameter of the intraductal lesion. In comparison with adjacent liver parenchyma, the density of tumor was divided as hyperattenuation, isoattenuation, or hypoattenuation in precontrast, arterial phase and portal venous phase. The presence of thickened bile duct walls was assessed in the portal venous phase by identifying the hilar bile duct wall thicker than 3 mm. The presence of enlarged perihilar and retroperitoneum lymph nodes was recorded when the short axis diameter of lymph nodes was greater than 10 mm. Vascular tumor thrombus were filing defects or cutoff in portal venous, hepatic venous and hepatic arterial systems. Intrahepatic bile duct calculus was round calcified shade in the dilated intrahepatic bile duct. Splenomegaly was identified when the outer edge of the spleen exceeded 7 rib elements.

### Statistical analysis

The patient demographics, clinical information, pathological information, preoperative radiologic diagnosis and CT image features of both kinds of tumor were compared.

Data management and analysis were performed using SPSS (version 19.0. for Windows, IBM-SPSS). A *p* value < 0.05 was considered to indicate a statistically significant difference.

## Results

### Patient demographics and clinical findings

58 HCC with HBDTTs (51 men and seven women; mean age, 49.8 years; range, 31–71 years) and 77 (49 men and 28 women; mean age, 58.6 years; age range, 29–80 years) were finally enrolled.

The incidence of HCC with BDTT was 5.1% (94/1827), while the incidence of HCC with HBDTT was 3.2% (58/1827). HBDTT was account for 61.7% (58/94) in HCC with BDTT.

On preoperative CT reports, there were 32 cases (55.2%) of HCC with HBDTT misdiagnosed as hilar CC. HCC with HBDTT was considered as the most likely diagnosis for 36.2% (21/58) of the HCC with HBDTT group. The remaining 8.6% (5/58) was reported as non-malignant lesions. In comparison, a diagnosis considering hilar CC was found in 96.1% (74/77) cases of the hilar CC group, while cholelithiasis with cholangitis and malignant tumor were considered for the remaining three cases.

The comparison of demographic and clinical features of HCC with HBDTT and hilar CC was shown in Table 1. Chronic hepatitis B and Elevated levels of AFP were more common in HCC with HBDTT group than that of hilar CC group (*p* <  0.001). On the other hand, elevated level of CA19–9 and CEA were more common in hilar CC group than that of HCC with HBDTT group (*p* <  0.001).

**Table 1:** Demographic and clinical features of HCC with HBDTT and Hilar CC

Clinical Information		HCC with HBDTT group (*n* = 58)	Hilar CC group (*n* = 53)	*p*
age		49.84 ± 10.23 (yrs)	58.61 ± 11.77 (yrs)	< 0.001
gender (male)		51(87.9)	49(63.6)	0.001
hepatitis history		42(72.4)	9(11.7)	< 0.001
jaundice		39(67.2)	60(85.7)	0.013
upper abdomen pain		39(67.2)	22(36.1)	0.001
serum tumor markers	AFP	39(67.2)	2(1.3)	< 0.001
	CA199	34(58.6)	66(85.7)	< 0.001
	CA125	11(19.0)	18(23.4)	0.537
	CEA	2(3.4)	23(29.9)	0.001

### Image analysis

**Fig. 2:**
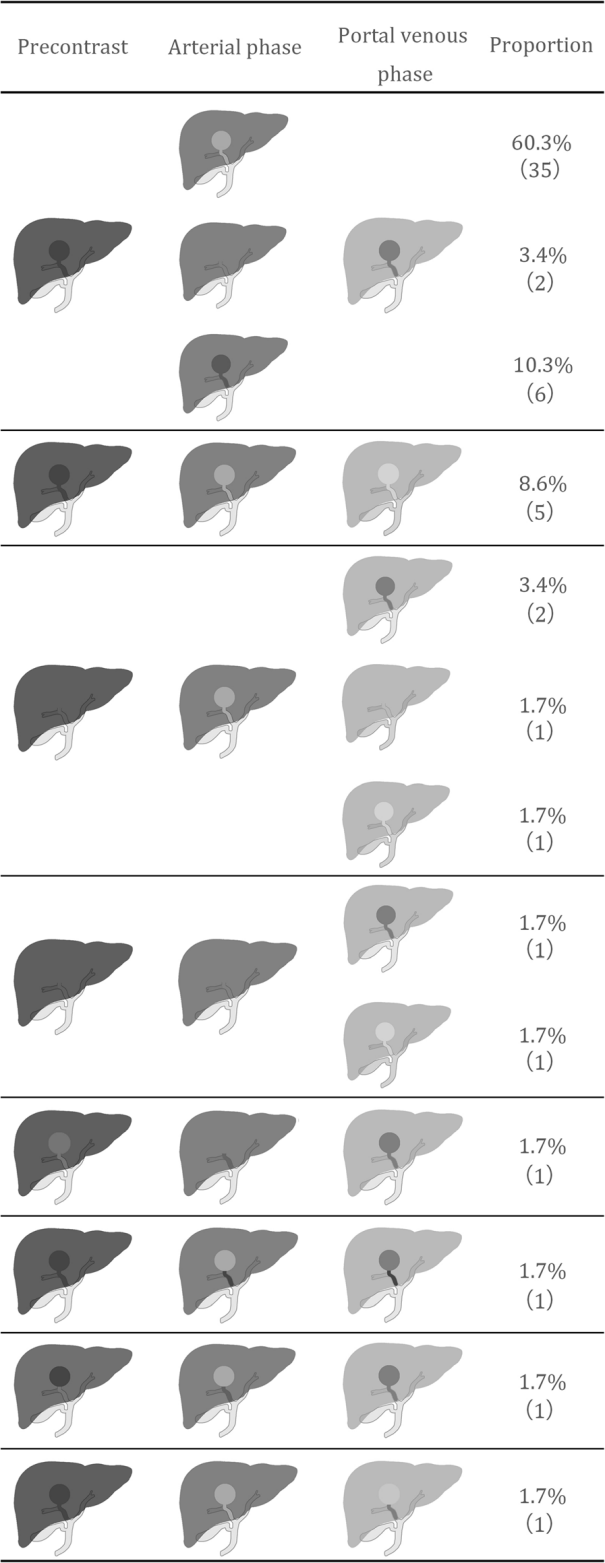
Different enhancement pattern of HCC with HBDTT. The last four lines showed different attenuation between HCC lesion and HBDTT

**Fig. 3:**
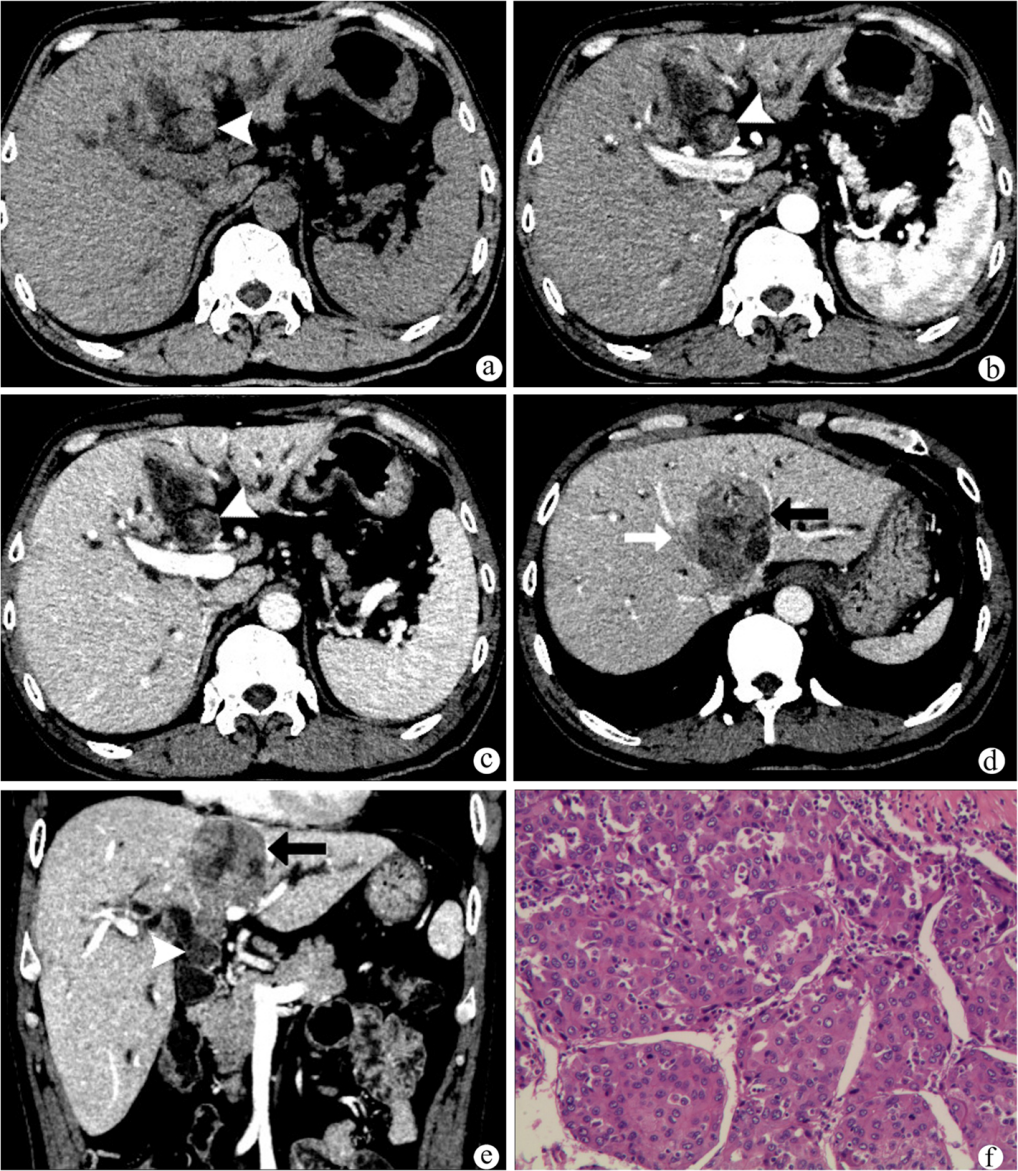
A patient with hepatocellular carcinoma(**a**-**f**). HBDTT (white arrow heads) show isoattenuation in plain CT image (**a**), heterogeneous enhancement with relative hyperattenuation in arterial phase (**b**) and hypoattenuation in portal venous phase(**c**), without bile duct wall thickening. The spleen is about eight rib elements (**a**-**c**). **d** There is a vascular tumor thrombus (white arrow) in the middle hepatic vein, which is hypoattenuation in portal venous phase. **e** A coronal image shows the relation between the intrahepatic hepatocellular carcinoma lesion (black arrow) and HBDTT (white arrow head). Diffused intrahepatic biliary dilation could be found (**a**-**d**). **f** (HE stain, original magnification× 200) The HCC is moderately differentiated trabecular type, grade II

**Fig. 4:**
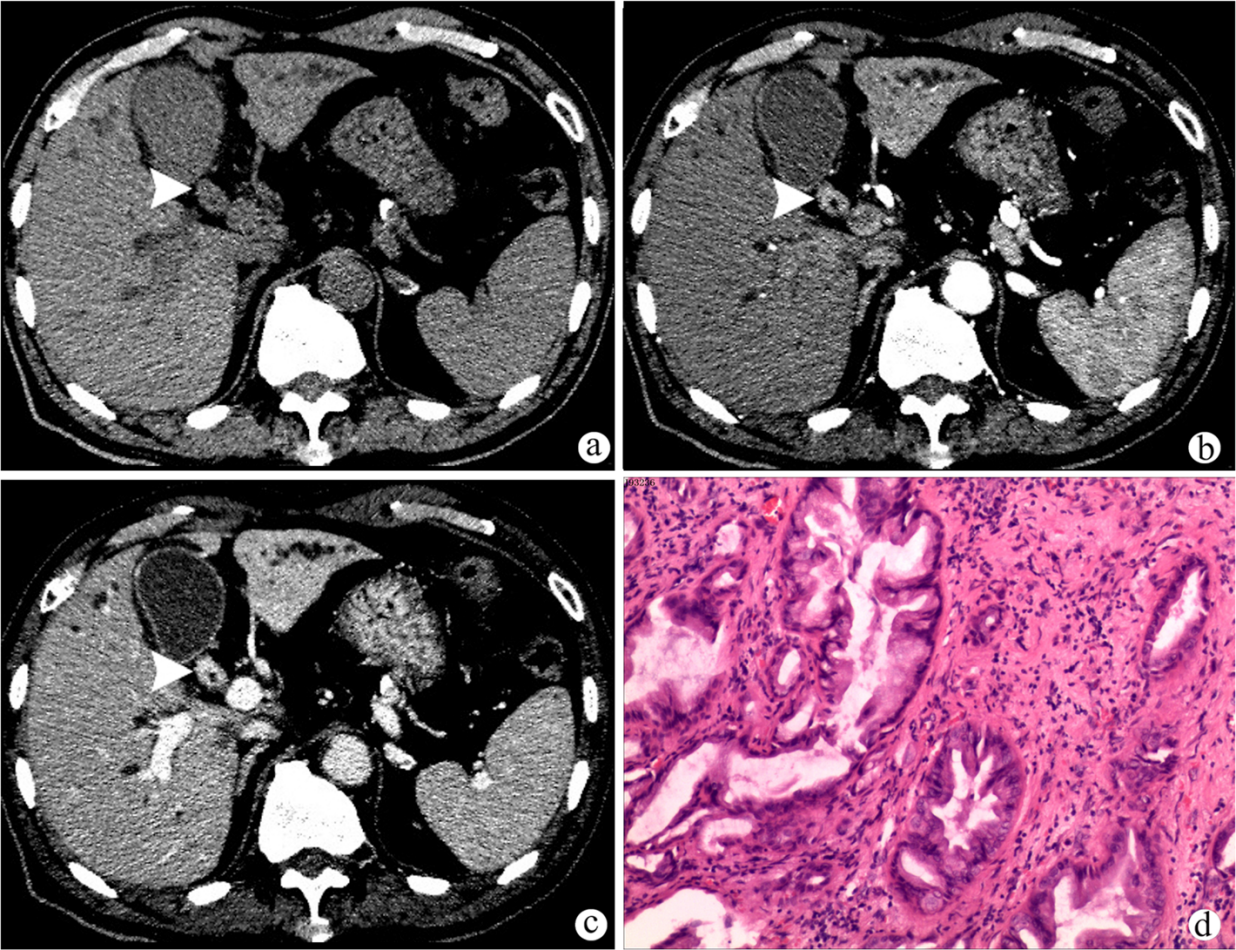
A patient with hilar cholangiocarcinoma (**a**-**d**). Hilar cholangiocarcinoma (white arrow heads) appears with thickened hilar bile duct wall, which shows hypoattenuation in plain CT image (**a**) and homogeneous enhancement with relative hyperattenuation in arterial phase (**b**) and portal venous phase (**c**). Diffused intrahepatic biliary dilation could be found (**a**-**c**). **d** (HE stain, original magnification× 40) The cholangiocarcinoma is grade II, accompanied with nerve invasion

The results of the CT findings in HCC with HBDTT and hilar CC were summarized in Table 2. The predominant enhancement pattern of HCC with HBDTT was hyperattenuation in arterial phase with washout in portal venous phase. Different enhancement patterns of HCC with HBDTT were shown in Figs. 2, 3 and Additional file 1: Figure S1, Additional file 2: Figure S2, and Additional file 3: Figure S3. Thickened and obviously enhanced hilar bile duct wall were more common in hilar CC group (Fig. 4) than that of HCC with HBDTT group.

**Table 2:** Comparison of CT findings between HCC with HBDTT and Hilar CC

Findings		HCC with HBDTT group (*n* = 58)	Hilar CC group (*n* = 77)	*p*
Tumor size (mm), mean ± SD		46.02 ± 27.28 (*n* = 58)	19.02 ± 10.55 (*n* = 62)	< 0.001
Parenchymal lesion with intraductal lesion		58(100)	14(18.2)	< 0.001
Intrahepatic bile duct dilation		58(100)	74(96.1)	0.259 (fisher)
CT density				
Precontrast	Hyperattenuation	1(1.7)	0	0.245 (fisher)
	Isoattenuation	6(10.3)	4(5.2)	
	Hypoattenuation	51(87.9)	73(94.8)	
Arterial phase	Hyperattenuation	47(81.0)	53(68.8)	0.111
	Isoattenuation	5(8.6)	17(22.1)	
	Hypoattenuation	6(10.3)	7(9.1)	
Portal venous phase	Hyperattenuation	8(13.8)	30(39.0)	< 0.001
	Isoattenuation	1(1.7)	42(54.5)	
	Hypoattenuation	49(84.5)	5(6.5)	
Thickened hilar bile duct wall		5(8.6)	72(93.5)	< 0.001
Vascular tumor embolus		26(44.8)	6(7.8)	< 0.001
Lymph node enlargement		7(12.1)	15(19.5)	0.248
Splenomegaly		20(34.5)	2(2.6)	< 0.001
Ascites		4(6.9)	0	0.068
Esophageal and gastric varices		2(3.4)	0	0.183 (fisher)
Calculus of intrahepatic bile duct		1(1.7)	11(14.3)	0.011

We selected the imaging findings that showed significant differences between HCC with HBDTT and hilar CC to calculate the sensitivity and specificity (Table 3). The criteria included parenchymal lesion with intraductal lesion, absence of hilar bile duct wall thickening, washout in portal venous phase, vascular tumor emboli and splenomegaly. Three of the five criteria (parenchymal lesion with intraductal lesion, washout in portal venous phase and normal hilar bile duct wall) showed high sensitivity (100, 91.4 and 84.5%) while all of the criteria showed high specificity (81.8–97.4%).

**Table 3:** Sensitivity and Specificity of the Significant Imaging Findings in the Diagnosis of HCC with HBDTT

CT findings	Sensitivity (*n* = 58 Lesions)	Specificity (*n* = 77 Lesions)
Parenchymal lesion with intraductal lesion	58(100)	63(81.8)
Unthickened hilar bile duct wall	53(91.4)	72(93.5)
Washout in portal venous phase	49(84.5)	72(93.5)
Vascular tumor embolus	26(44.8)	71(92.2)
Splenomegaly	20(34.5)	75(97.4)

Note—Data are the number of lesions, with the sensitivity and specificity percentages in parentheses

Sensitivity refers to the proportion of the number of correctly diagnosed HCC with HBDTTs to that of all HCCs

Specificity refers to the proportion of the number of correctly diagnosed hilar CCs to that of all hilar CCs

A score system consisted of the five parameters mentioned above was trialed to facilitate the diagnosis of HCC with HBDTT. One point is allocated to each of the following if present on imaging: the presence of parenchymal lesion with intraductal lesion, non-thickened hilar bile duct wall, hypoattenuation in portal venous phase, vascular tumor embolus or splenomegaly. The total score ranges from 0 to 5 points where a total score of 2 or more showed, accuracy, sensitivity and specificity of 96.39, 100 and 92.5% respectively in diagnosing HCC with HBDTT.

### Treatment and follow up of the patients

The number of patients underwent simple hepatectomy was 36, and the number of hepatectomy plus bile duct excision was 11. One of the HCC with HBDTT patients only received tumor biopsy instead of resection.

**Fig. 5:**
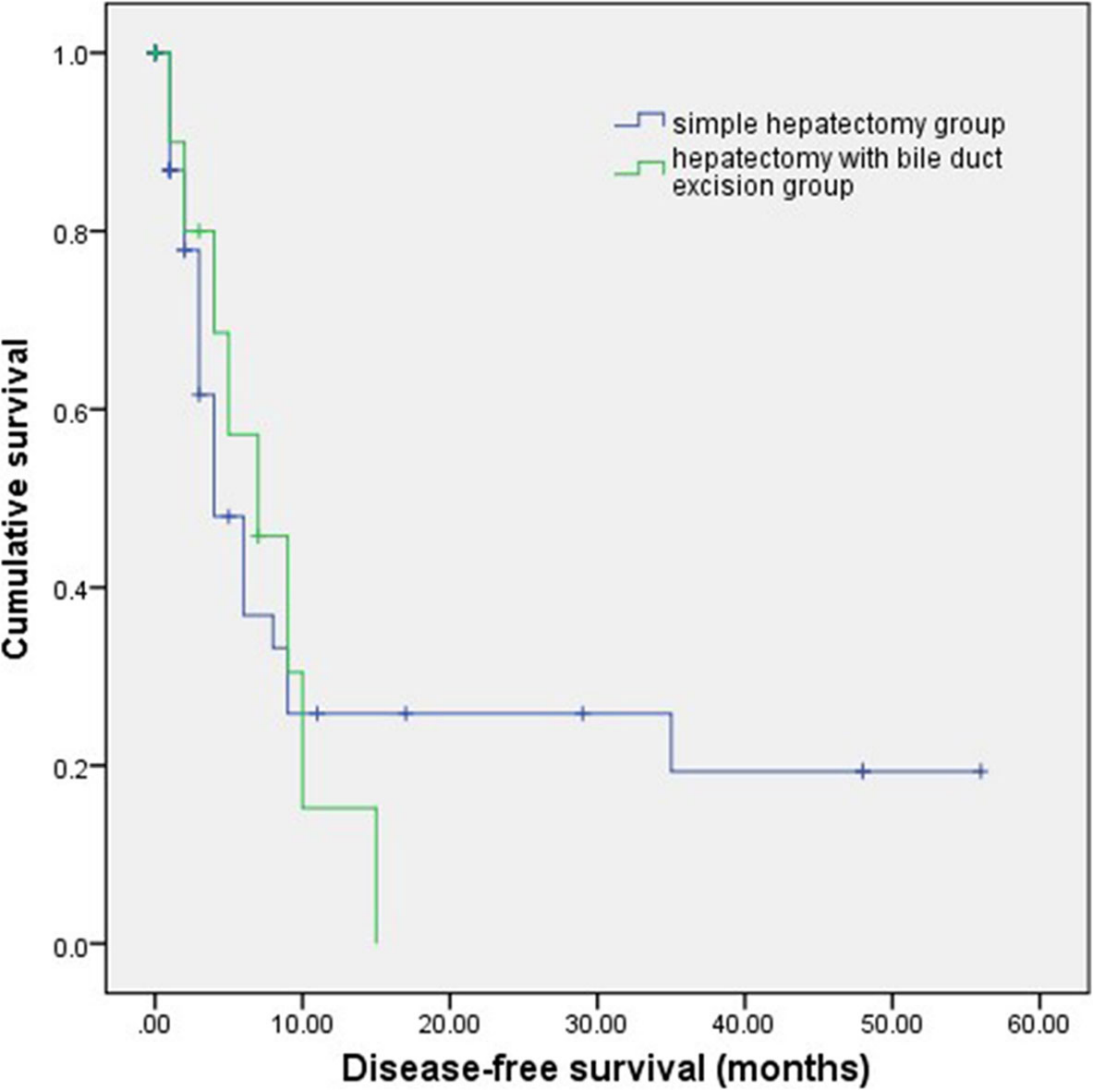
Disease-free survival curve for 46 simple hepatectomy patients and 11 hepatectomy with bile duct excision patients (log-rank test, *p* = 0.88)

Disease-free survival (DFS) was defined as the interval between the date of surgical resection and diagnosis of recurrence or the most recent follow-up date. The last observation (censoring date) in this study was made on November 30, 2019. The median follow-up of the 57 patients was 14.4 months. The DFS of simple hepatectomy group was 16.1(8.2~23.9) and the DFS of hepatectomy with bile duct excision group was 7.3(4.2~10.4). There was no statistically significant difference between the two groups (*p* = 0.88). The survive curve was shown in Fig. 5.

## Discussion

Although HCCs are particularly common, HCC with HBDTTs are relatively rare, accounting for about 0.53 to 12.9% of HCCs [2–6]. The incidence of HCC with BDTT was 5.1 and 3.2% for HCC with HBDTT in our study, which is consistent with previous reports. Moreover, we found 55.2% of HCC with HBDTTs misdiagnosed as hilar CCs on preoperative CT scan. HCC with HBDTTs share several image features with hilar CCs where radiological diagnosis remains challenging. As different surgical treatments are required, differentiating between these two diseases is of vital importance. There are, however, some helpful features to distinguish HCC with HBDTTs from hilar CCs that were found in our study, including a younger male predominance with a history of chronic hepatitis B infection, upper abdomen pain, elevated level of AFP, washout in portal phase, normal thickness of hilar bile duct wall without abnormal enhancement, vascular tumor embolus, enlargement of lymph nodes and splenomegaly. On the other hand, symptoms of jaundice, increased level of CA19–9 and CEA, intrahepatic bile duct calculus, as well as thickened bile duct wall on CT images favors the diagnosis of hilar CCs over HCC with HBDTTs. Moreover, the score system provides multiple factor analysis, where a total score of two or more is highly suggestive for a diagnosis of HCC with HBDTT.

Previous study illustrated several possible pathogeneses for HCC with HBDTT as follow [9, 13]: (1) intrahepatic primary HCC lesion directly invades the adjacent bile duct and with intraluminal extension to the hilar bile ducts; (2) the tumor tissue ruptures after invasion of adjacent bile duct, then tumor tissue that depart from the primary HCC lesion migrates to the hilar bile duct to form a HBDTT; (3) hemorrhage in the biliary tract close to the primary HCC tumor fills the bile duct with cancer-containing blood clots to varying degree. In our study, 56/58 HBDTT lesions were directly connected to the intrahepatic HCC lesion, probably in favor of the first pattern. The other two HBDTTs were hemorrhage presenting as hyperattenuation in plain CT scan, without enhancement in post-contrast phase. However, the second growth pattern of HBDTT was not found in our study, most likely due to the low incidence rate.

It is well known that chronic hepatitis B virus and hepatitis C virus infection is the leading cause of liver cirrhosis, and liver cirrhosis had been proved to be the foremost clinical risk factor for development of HCC [17]. So the history of chronic hepatitis B infection appears to be important for HCC with HBDTTs. As a manifestation of cirrhosis, splenomegaly is very common in the HCC with HBDTT group of our study. In comparison, Hepatitis B and C virus were suggested to be risk factors for intrahepatic cholangiocarcinoma [18, 19]. However, our study did not demonstrate significant correlation with hilar CC. Much possible risk factors for hilar CCs such as hepatobiliary flukes, primary sclerosing cholangitis, choledocholithiasis and hepatolithiasis had been analyzed [20–22]. Only a few choledocholithiasis and hepatolithiasis were observed in our study, but the cause-result order remains uncertain.

Though the sensitivity and specificity of AFP for diagnosis of HCC is not satisfied [23], the elevation of AFP is useful in the differentiation of hilar CCs. Elevation of CA19–9 in both HCC with HBDTTs and hilar CCs are not rare [8, 24]. As we all know, CA19–9 can be secreted by biliary tract epithelial cells physiologically [25]. When the drainage of intrahepatic bile duct system is obstructed by either benign or malignant causes, CA19–9 in the bile mucin may permeate into serum and lead to the non-specific elevation of CA19–9 in serum [25, 26]. Therefore, elevated CA19–9 level lacks specificity in differential diagnosis on this occasion.

In our study, more than half of HCC with HBDTTs were misdiagnosed as CCs originated from hilar bile duct and grew as a mixed type. Like most previous studies, we also found that HCC with HBDTTs are always observed with both parenchyma and intraductal lesions [2, 3]. But it will be extremely difficult when the lesion in liver parenchyma is small or even “invisible” [24, 27, 28]. However, Liu et al. claimed that CT and MR are useful for detection and diagnosis of small HCC with BDTT [29]. Therefore, distinctive image features of HBDTTs seem especially important to be recognized. HCC has increased arterial blood supply [17], so it is usually hyperattenuation in arterial phase, and comparatively hypoattenuation in portal venous phase. Most HBDTT shows the same enhancement pattern as well, because it shares the same blood supply as it is always directly connected to HCC. However, some HCCs show iso- or hypoattenuation in arterial phase instead of “fast in” sign. Previous study has suggested a correlation between the enhancement of HCC and the degree of tumor differentiation [30], the percentage of necrotic tissue and the speed of CT scanning. So hypoattenuation in portal venous phase seems to be more important for identifying HCCs these days.

What’s more, HCC can invade into bile duct and grow through or directly invade the hilar bile duct to form HBDTT, accompanied with necrosis and hemorrhage [9, 24]. The reinforcement level of HBDTT is inversely proportional to component of necrosis and blood clots. However, HBDTT seldom infiltrate into bile duct wall [11, 31], so hilar bile duct is often regular or even thinner due to extreme dilation and has no extraordinary enhancement. In comparison, the most common morphological type of hilar CC is periductal-infiltrating type [32]. It usually manifests a narrowed hilar bile duct with irregular wall thickening or sometimes obliterated, which typically shows progressively delayed enhancement [15, 33]. A combination of the periductal and mass-forming mixed types could also be found in hilar area. The difficulty for differential diagnosis is to identify HCC lesions from mass-forming CCs. However, the key points are wash out in portal venous phase and the presence of tortuous tumoral vessels [34, 35].

Non-specific lymph node enlargement of HCC with HBDTT and hilar CC was similar. However, there was statistically significant differences in pathological proven lymph node metastasis between the two groups. Perihepatic lymph nodes enlargement was proved to be associated with fibrosis and hepatocellular injury [36]. A majority of enlarged lymph nodes in HCC are benign, probably closely related to background fibrosis. The incidence of lymph node metastasis, also known as malignant lymph node enlargement, has been reported to be 1.2–1.4% in HCC after hepatectomy [37, 38] compared to 43.4–52.7% in hilar CC [39, 40]. Surprisingly, our study found 12.5% lymph node metastasis in HCC with HBDTT, which is higher than those reported in previous literatures. Some studies had mentioned higher incidence of lymphovascular invasion in HCC with BDTT than HCC without BDTT [41, 42]. This finding is unexpected and may suggest that HCC with HBDTT is more aggressive via lymphatic metastasis.

HCC with HBDTTs were always combined with vascular tumor embolus, especially in portal venous, indicating poor prognosis [42]. Several studies have found that portal vein invasion in HCC with BDTT was much higher than HCC without BDTT [2, 8]. This may also suggest that HCC with BDTT are more invasive than HCC without BDTT.

Thus far, several studies have explored the component and features about HCC with BDTT [5, 8, 17]. Many surgeons reached a consensus that hepatectomy for HCC with HBDTT is necessary [10, 11, 43, 44]. In comparison, bile duct resection in patients with HCC and HBDTT remains controversial. Although some studies proposed that bile duct resection has better outcome [12, 42], most studies indicated that bile duct resection is not necessary, because BDTT is not adhered to and seldom infiltrated into the bile duct wall [11, 24, 31]. As the surgical procedure is totally different for HCC with HBDTT and hilar CC, it is of vital importance to differentiate them preoperatively and give reliable information to aid surgical planning. In fact, clinical misdiagnosis of HCC with HBDTT is not rare. The reason, on the one hand, is that radiologists lack sufficient awareness of the characteristics of HCC with HBDTT. On the other hand, there are limited literatures that described features that aid the differentiation of HCC with HBDTT from hilar CC. But in our study, the important features for differentiation with hilar CC are explained. Jung et al. compared several CT image features between HCC with HBDTT and intraductal papillary cholangiocarcinoma [45]. However, to our knowledge, there was no previous study describing the differentiating characteristics of HCC with HBDTT and hilar CC on CT scan.

Several limitations to this study need to be acknowledged. First of all, selection bias could not be fully avoided in this retrospective study. We have included patients diagnosed with HCC with HBDTT based on pre-operative enhanced CT scan followed by histopathological confirmation. Those patients who were diagnosed by gadoxetic acid–enhanced liver MRI without histopathological confirmation were excluded. Second, the size of patient included were limited due to various incomplete data. Although our study included as much HCC with HBDTTs as possible, future study with larger HCC with HBDTTs population needs to be conducted. Thirdly, the delayed phase images, which are known to be more useful in differentiating HCC with HBDTTs from hilar CCs [46], were not considered in our routine CT scan for abdomen. However, most routine abdominal CT scan do not contain the delayed phase. Lastly, MRI displays more detailed information for the diagnosis with HCC with HBDTTs than hilar CCs. However, this retrospective study only focused on CT findings due to its higher use. Further studies exploring the MRI imaging features may be useful to aid the differentiation between HCC with HBDTTs and hilar CCs.

## Conclusions

In conclusion, HCC with HBDTT can be distinguishable from hilar CC using enhanced CT scan. The diagnosis of HCC with HBDTT is favored when the lesion demonstrate involvement of both liver parenchyma and hilar bile duct with background hepatitis B infection, especially when it demonstrates hypoattenuation during the portal venous phase. Other characteristics such as presence of dilated distal bile duct or vascular tumor embolus, the absence of thickened bile duct wall, splenomegaly and elevation of serum AFP level is also supportive of the diagnosis.

## Supplementary information

**Additional file 1: Figure S1**. A patient with hepatocellular carcinoma (**a**-**e**). HBDTT (white arrow heads) appeared like irregular bile duct wall thickening, show hypoattenuation in plain CT image (**a**), enhancement with relative hyperattenuation in arterial phase (**b**) and heterogeneous hypoattenuation in portal venous phase (**c**). **d** A coronal image shows the connection of the intrahepatic HCC lesion (white arrow) and HBDTT (white arrow head), both show hypoattenuation in portal venous phase. Intrahepatic biliary dilation could be found (**a**-**c**). **e** (HE stain, original magnification×40) The thrombi do not adhere to the bile duct wall, without bile duct infiltration and mainly consisted of tumor nests.

**Additional file 2: Figure S2**. A patient with hepatocellular carcinoma (**a**-**d**). Intrahepatic HCC lesion (black arrows) and HBDTT (white arrow heads) show hyperattenuation in plain CT image (**a**). The HCC lesion show relative isoattenuation in arterial phase (**b**) and hypoattenuation in portal venous phase(**c**). The HBDTT show hypoattenustion without enhancement in both two phase (**b**-**c**). The spleen is about nine rib elements (**a**-**c**). **d** (HE stain, original magnification×200) The HCC is moderately differentiated trabecular type, grade II, and part of the lesion was clear cell type.

**Additional file 3: Figure S3**. A patient with hepatocellular carcinoma (**a**-**d**). HBDTT (white arrows) show hypoattenuation in plain and postcontrast CT images with increased CT value (**a-c**). The spleen is more than seven rib elements (**a**-**c**). **d** (HE stain, original magnification×40) The HCC is moderately differentiated trabecular type, grade II.

## Data Availability

Original data and material can be available from the corresponding author if. necessary.

## Abbreviations

AFP: Alpha fetoprotein
BDTT: Bile duct tumor thrombus
CA-125: Carbohydrate antigen 125
CA19–9: Carbohydrate antigen 19–9
CEA: Carcinoembryonic antigen
CT: Computed tomography
HBDTT: Hilar bile duct tumor thrombus
HCC: Hepatocellular carcinoma
Hilar CC: Hilar cholangiocarcinoma
MRI: Magnetic Resonance Imaging
