# Supplementary figures and images for: Hepatocellular carcinoma with hilar bile duct tumor thrombus versus hilar Cholangiocarcinoma on enhanced computed tomography: a diagnostic challenge
Source: BMC Cancer. 2020 Jan 22;20:54. doi: 10.1186/s12885-020-6539-7 (PMC6977349; doi:10.1186/s12885-020-6539-7)

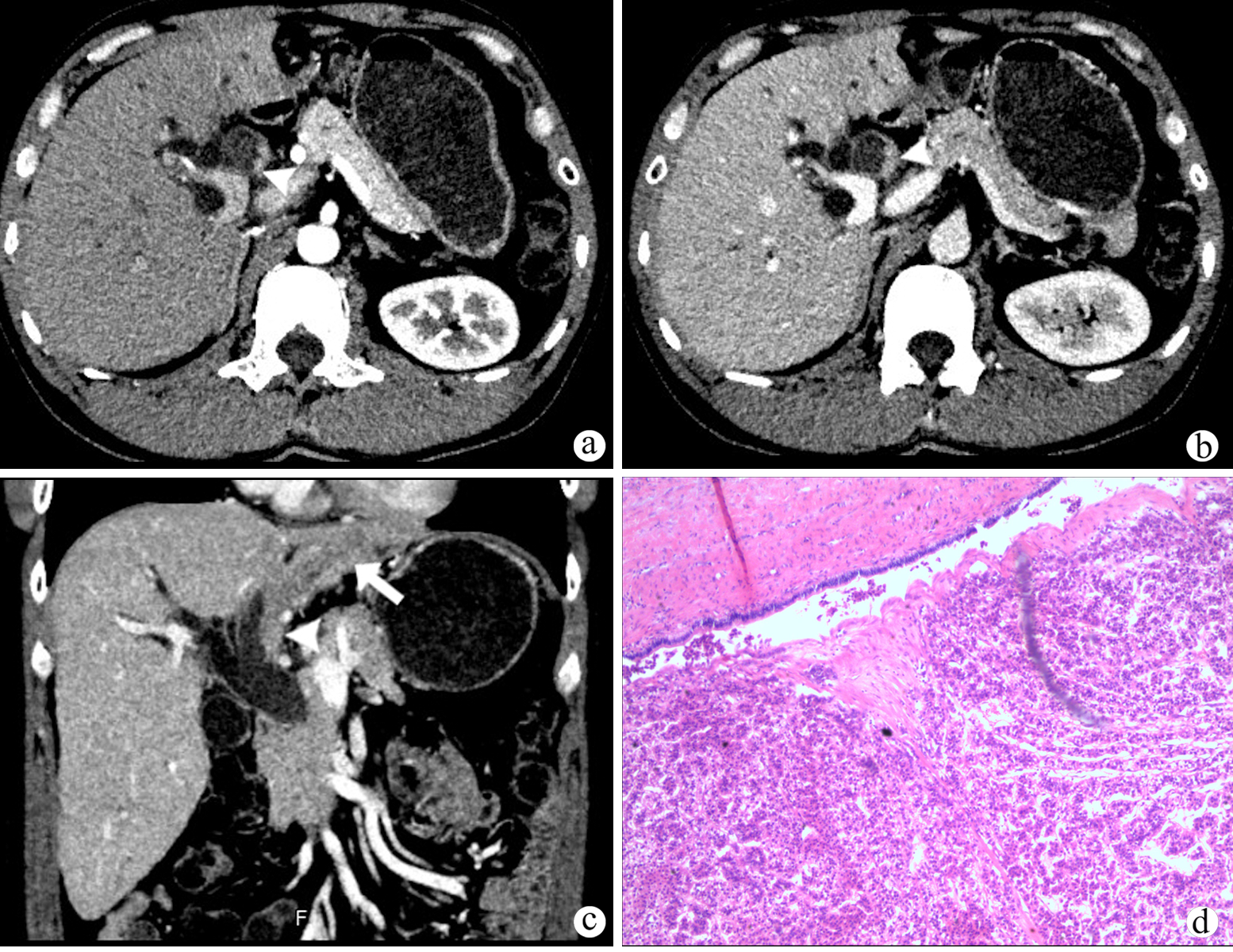

Supplement: Supplementary file 1 — Additional file 1: Figure S1. A patient with hepatocellular carcinoma (a-e). HBDTT (white arrow heads) appeared like irregular bile duct wall thickening, show hypoattenuation in plain CT image (a), enhancement with relative hyperattenuation in arterial phase (b) and heterogeneous hypoattenuation in portal venous phase (c). d A coronal image shows the connection of the intrahepatic HCC lesion (white arrow) and HBDTT (white arrow head), both show hypoattenuation in portal venous phase. Intrahepatic biliary dilation could be found (a-c). e (HE stain, original magnification×40) The thrombi do not adhere to the bile duct wall, without bile duct infiltration and mainly consisted of tumor nests. [file 12885_2020_6539_MOESM1_ESM.tif]

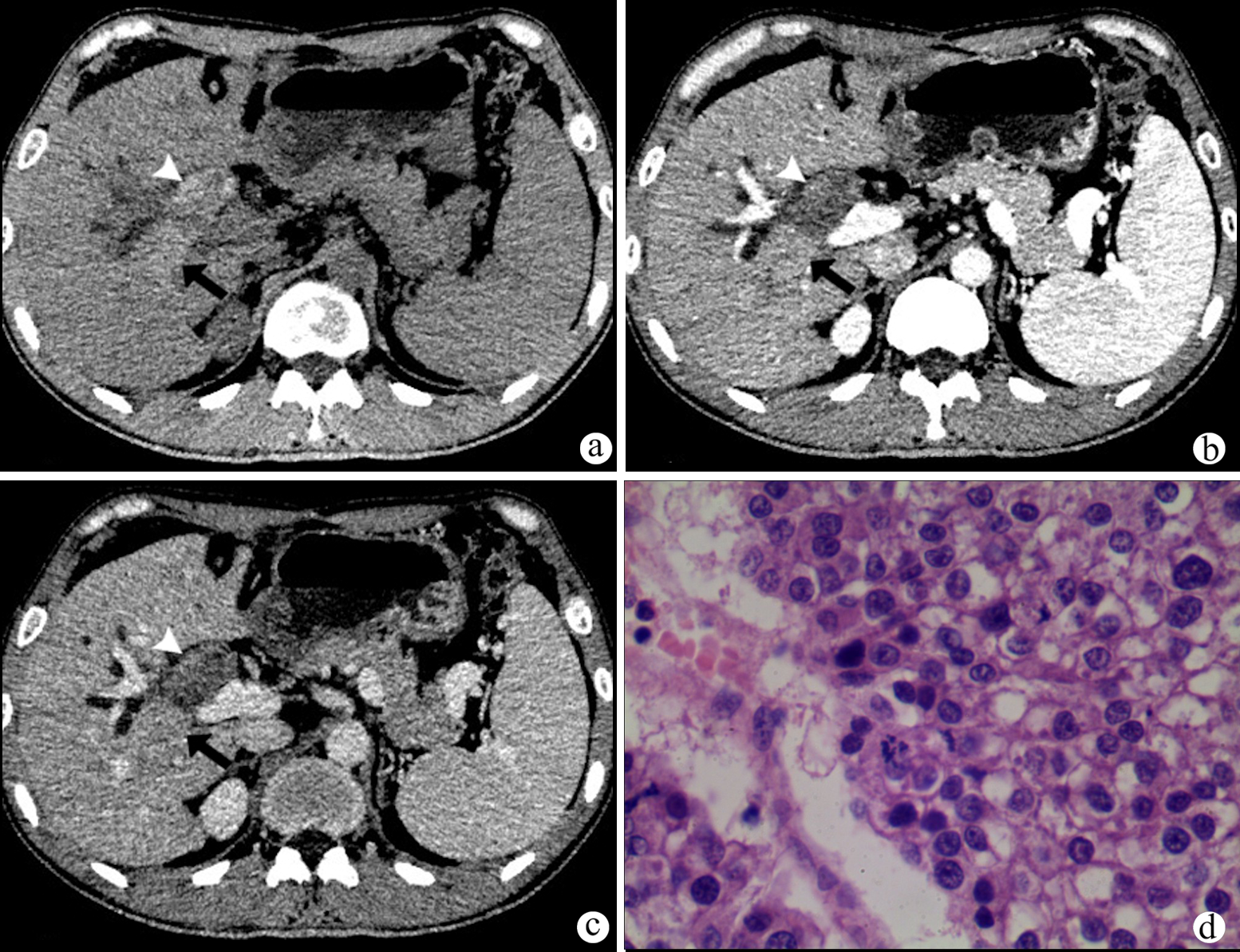

Supplement: Supplementary file 2 — Additional file 2: Figure S2. A patient with hepatocellular carcinoma (a-d). Intrahepatic HCC lesion (black arrows) and HBDTT (white arrow heads) show hyperattenuation in plain CT image (a). The HCC lesion show relative isoattenuation in arterial phase (b) and hypoattenuation in portal venous phase(c). The HBDTT show hypoattenustion without enhancement in both two phase (b-c). The spleen is about nine rib elements (a-c). d (HE stain, original magnification×200) The HCC is moderately differentiated trabecular type, grade II, and part of the lesion was clear cell type. [file 12885_2020_6539_MOESM2_ESM.tif]

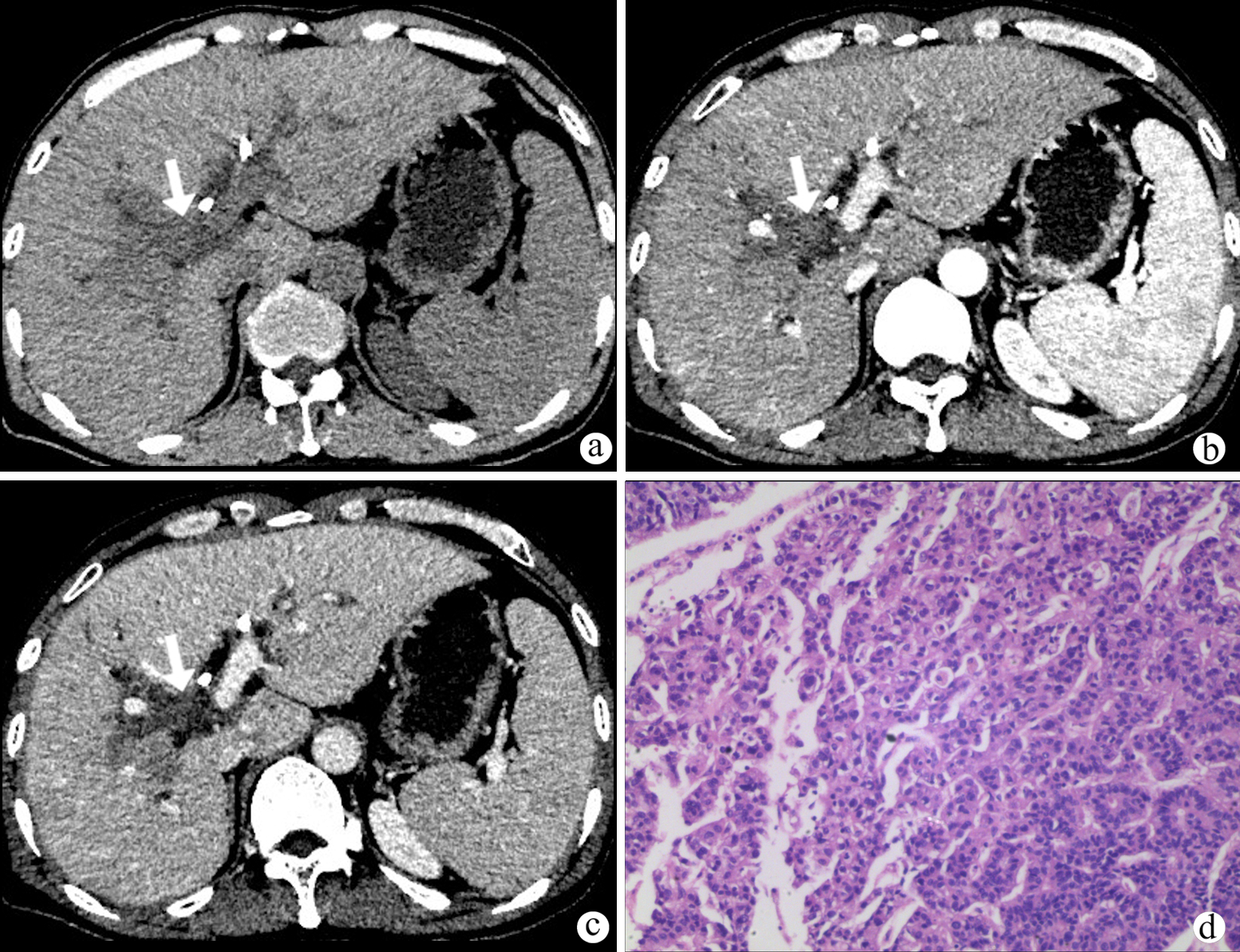

Supplement: Supplementary file 3 — Additional file 3: Figure S3. A patient with hepatocellular carcinoma (a-d). HBDTT (white arrows) show hypoattenuation in plain and postcontrast CT images with increased CT value (a-c). The spleen is more than seven rib elements (a-c). d (HE stain, original magnification×40) The HCC is moderately differentiated trabecular type, grade II. [file 12885_2020_6539_MOESM3_ESM.tif]
